# Diagnostic significance of noncoding RNAs in kawasaki disease: A systematic review and meta-analysis

**DOI:** 10.3389/fped.2022.1071434

**Published:** 2023-01-16

**Authors:** Xiaoling Zhong, Xufeng Jia, Hui Wang, Guihua Chen, Hongxia Li, Pingping Li, Taoyi Yang, Jiang Xie

**Affiliations:** Department of Pediatrics, The Third People's Hospital of Chengdu/The Affiliated Hospital of Southwest Jiaotong University, Chengdu, China

**Keywords:** noncoding RNA, kawasaki disease, diagnosis, biomarker, meta-analysis

## Abstract

**Objective:**

Kawasaki disease (KD) is a systemic vasculitis disease, and early effective intervention would reduce the occurrence of coronary artery lesions (CALs). Recently, many scholars have been committed to studying the relationship between noncoding RNAs and KD. This systematic review aimed to analyze the diagnostic value of noncoding RNAs(ncRNAs) in distinguishing different KD status.

**Methods:**

We searched for the literature about diagnostic values of ncRNAs in KD in CNKI, VIP, Wanfang, China Biomedical Literature Database as well as PubMed, Web of Science, Embase, and Cochrane Library up to April 15, 2022. All included studies were further analyzed using STATA 12.0, Meta-disc 1.4 and RevMan 5.4 software.

**Results:**

A total of six studies investigating the diagnostic performance of ncRNAs in differentiating KD-CAL (*n* = 101) from KD-NCAL patients (*n* = 123) were included in this this meta-analysis. The calculated area under the curve(AUC) was 0.83 (0.80–0.86). Four studies on the diagnostic performance of ncRNAs in differentiating acute KD patients (*n* = 139) from convalescent KD patients (*n* = 109) were included. The calculated AUC was 0.87 (0.84–0.90). Four studies focused on the diagnostic performance of ncRNAs combined with other laboratory indexes in KD by assessing 137 KD patients and 152 febrile controls. The calculated AUC was 0.90 (0.87–0.92). Four studies assessed the diagnostic performance of ncRNAs in differentiating intravenous immunoglobulin (IVIG)-resistant KD patients from IVIG-responsive KD patients. The calculated AUC was 0.9135 ± 0.0307. These results indicated that ncRNAs have a good diagnostic efficacy in KD.

**Conclusions:**

This meta-analysis showed that ncRNAs have potential as a biomarker for distinguishing different KD status. However, since limited studies were included in this meta-analysis, larger and well-designed diagnostic studies should be conducted to validate these results.

**Systematic Review Registration:**

INPLASY.COM, identifier: doi: 10.37766/inplasy2022.10.0035.

## 1. Introduction

Kawasaki disease (KD) is an acute, self-limited febrile illness of unknown cause that mainly affects children <5 years old and is the most common cause of acquired heart disease in children (1). It is associated with vasculitis of medium-sized arteries, particularly affecting the coronary arteries (2). Approximately 25% of untreated and 4% of treated patients experience coronary artery lesions (CALs) (2). The diagnosis of Kawasaki disease is confusing, especially in the situation of atypical KD. Due to the lack of specific laboratory biomarkers, the diagnosis of KD is still mainly based on clinical manifestations. This method is not completely reliable, and it is problematic due to the time-sensitive nature of the disease. Coronary artery lesions cannot be detected promptly and not treated timely may have significant long-term implications for KD patients (3). Therefore, it is important to establish a set of sensitive, specific and reproducible laboratory markers for detecting KD-CAL. The intravenous immunoglobulin (IVIG)-resistant KD patients are more likely to develop CAL (4). The early prediction of the response to immunoglobulin of KD patients allows clinicians to make a therapy plan in advance and conduct more aggressive treatment to prevent the occurrence of CAL. Japanese scholars have constructed a risk scoring system for predicting KD-IVIG resistance (5, 6). However, it's not accurate enough for clinical application in countries other than Japan (7). Therefore, better predictive biomarkers need to be developed for widespread clinical application. Early laboratory biomarkers could predict complications, and well-timed therapy could reduce adverse outcomes. Therefore, finding highly sensitive and specific biological indicators that can identify coronary artery damage in KD as early as possible may help to identify the optimal timing of treatment and prevent or treat KD-CAL effectively by blocking disease progression.

Noncoding RNAs (ncRNAs) include short RNAs represented by microRNAs (miRNAs), small interfering RNAs (siRNAs), small nuclear RNAs (snRNAs) and long noncoding RNAs (lncRNAs) (8). In the past 20 years, the discovery of ncRNAs and the resulting research have greatly changed the understanding of RNA fractions. NcRNAs not only play an auxiliary role as intermediate carriers of inherited information but also exert complex and precise functions in development and gene expression regulation (9). A previous study indicated that ncRNAs play key roles in regulating gene expression in cardiovascular phylogeny, physiology and disease physiology (10). Several studies have demonstrated that ncRNAs have the potential to be biomarkers for risk stratification, diagnosis and prognosis of cardiovascular diseases (11, 12). A previous study found that miRNAs are readily detectable in the plasma and protected from RNase degradation by sequestration, suggesting that miRNAs are suitable as ideal biomarkers (13). The role of miRNAs in KD with CAL patients has been widely studied. Wang YF et al., for example, found that the miRNA let-7i-3p can be used as a potential biomarker for KD-CAL patients (14). Wu R et al. reported that miR-186 was upregulated in serum in acute KD patients and appeared to be highly specific to the acute phase of KD (15). In addition, miRNAs and lncRNAs also play important roles in the pathological process of KD with CAL. For instance, the lncRNA XLOC_006277 was overexpressed in patients with acute KD and decreased in IVIG-responsive patients (16). These studies show that ncRNAs may be used as potential biomarkers for the diagnosis of KD.

A previous systematic review/meta-analysis found that the neutrophil-to-lymphocyte ratio could be used as a biomarker in IVIG-resistant KD (17), and another meta-analysis studied the diagnostic significance of miRNAs in KD (18). Several recent studies have focused on the diagnostic accuracy of ncRNAs for KD-CAL and their value in differentiating the acute phase of KD and convalescent KD (12, 19–23). However, none of the systematic reviews/meta-analysis studied the diagnostic accuracy of ncRNAs in KD-CAL or the accuracy in differentiating the acute phase of KD and convalescent KD patients. Therefore, we collected all published case‒control studies to explore the diagnostic accuracy of the ncRNAs for distinguishing different KD statuses, including differentiating KD-CAL from KD-NCAL, the acute KD from convalescent KD patients, IVIG-resistant KD from IVIG-responsive KD and the diagnostic accuracy of miRNAs combined with other clinical laboratory tests in KD.

## 2. Materials and methods

This systematic review/meta-analysis was reported in accordance with the Preferred Reporting Items for Systematic Reviews and Meta-Analyses (PRISMA) Statement (24).

### 2.1. Search strategy

We initially identified domestic and foreign studies focusing on noncoding RNA as a diagnostic marker of KD by searching the PubMed, Web of Science, Embase and Cochrane Library databases, CNKI, VIP, China Biology Medicine disc databases and Wanfang databases until April 15, 2022. The following search terms were used: (1) “Kawasaki disease” or “mucocutaneous lymph node syndrome”; (2) “non-coding RNA” or “noncoding RNA” or “ncRNA” or “miRNA”, “microRNA” or “siRNA” or “snoRNA” or “circRNA” or “lncRNA” or “RNA”. These search terms were combined using the conjunction “and”. The language was restricted to English or Chinese. This meta-analysis has been registered on the International Platform of Registered Systematic Review and Meta-analysis Protocols (INPLASY2022100035).

### 2.2. Study selection

The criteria for inclusion were as follows:

(1) Studies about the diagnostic performance of ncRNAs in KD patients.
(2) All patients were diagnosed with consistent KD criteria.

The criteria for exclusion were:

(1) Duplicate studies, editorials, reviews, conference articles, expert opinions, abstracts, or case reports without controls.
(2) No complete data to form a 2 × 2 table.
(3) Only focus on the diagnostic values of ncRNAs in differentiating acute KD patients without any complications from healthy controls/febrile controls.

### 2.3. Data collection and assessment of research quality

Two reviewers independently extracted the following data for each eligible study: first author's last name, year of publication, specimen, number of cases and controls, age and dysregulated ncRNAs. Any disagreements were resolved by consensus. Methodological quality of the included studies was conducted independently by two researchers using the QUADAS-2 checklist, which consisted of 14 items, including 4 important domains: patient selection, index test, reference standard, flow and timing (25).

### 2.4. Statistical analysis

Cochran's Q test and the *I*^2^ test were used to evaluate the heterogeneity among the included studies (26). If significant heterogeneity existed (*I*^2^ > 50%), then the random effects model was used; otherwise, the fixed effects model was used to calculate the results. If significant heterogeneity was identified, sensitivity analysis was performed to detect whether a single study had significantly affected the pooled results by removing one study in each turn. Potential publication bias was assessed by the Deeks test. STATA 12.0 (College Station, TX), MetaDisc 1.4(Spain) and RevMan Manager 5.4 (Cochrane, Oxford, UK) were employed to conduct all statistical analysis.

## 3. Results

**Figure 1:**
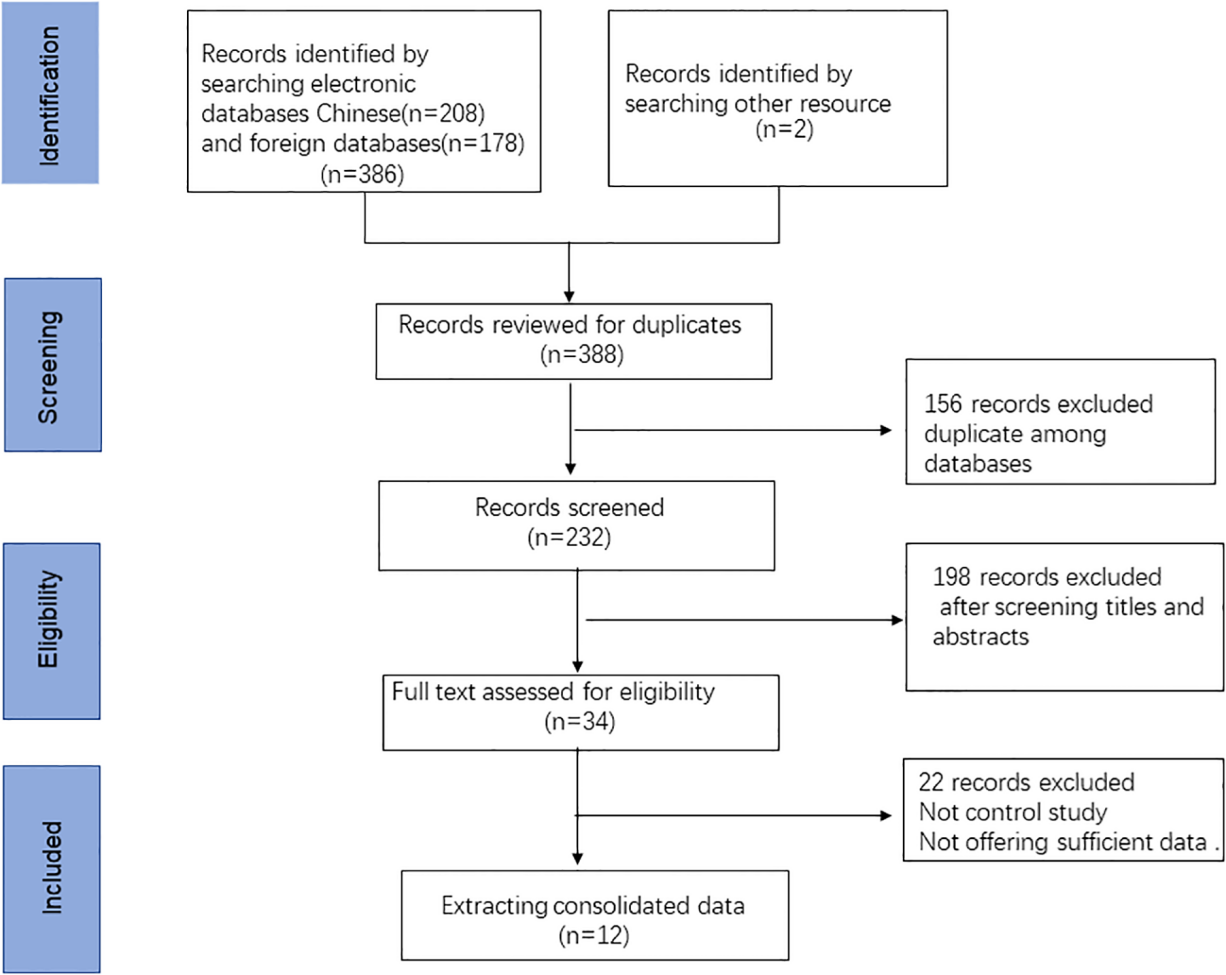
A flow diagram demonstrating the study selection process.

In total, we retrieved 386 articles, and another 2 articles were found after screening the references. Of these, 156 duplicates were excluded, and 232 different records remained. Then, after screening the abstracts and titles, 198 studies were excluded for the following reasons: reviews, abstracts and nonhuman articles. Subsequently, 22 articles were excluded after full text screening. Finally, 12 articles containing 18 studies were eligible for inclusion in this systematic review and meta-analysis (12, 19–23, 27–32). The flow diagram of the selection process of this meta-analysis is presented in Figure 1. Of those included studies, a total of 5 studies focused on the diagnostic value of ncRNAs in KD-CAL. A total of 4 articles assessed the value of ncRNAs combined with other laboratory indicators for the diagnosis of KD patients. A total of 4 articles assessed the diagnostic values of ncRNAs in differentiating acute KD patients from convalescent KD patients. A total of 2 articles assessed the value of ncRNAs in IVIG-resistant KD patients.

### 3.1. Study characteristics and quality assessments

**Figure 2:**
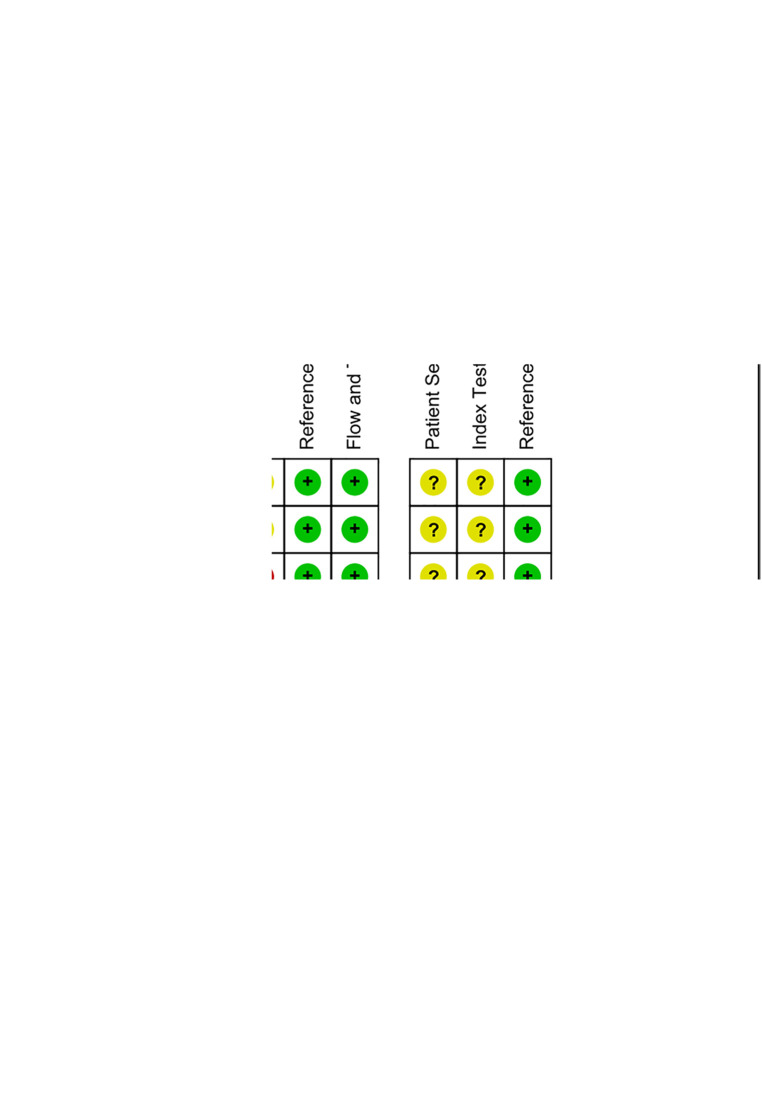
(**A**) risk of bias and applicability concerns graph: reviews the judgments of the author about each domain presented as percentages across included studies. (**B**) Risk of bias and applicability concerns summary: reviews judgments of the author about each domain for each included study.

The studies included in this meta-analysis were all about the diagnostic accuracy of ncRNAs, mainly miRNAs, in differentiating KD-CAL patients from KD-NCAL patients, acute KD patients from convalescent KD patients, IVIG-resistant KD patients from IVIG-responsive KD patients and the diagnostic values of ncRNAs combined with other laboratory indexes in KD patients. Those studies were published from 2015 to 2021 in China. The characteristics of the included studies are presented in Table 1. In those studies, there were 101 KD-CAL patients and 123 KD-NCAL patients (Tables 1, 2). There were 139 acute KD patients and 109 convalescent KD patients. There were 246 IVIG-responsive KD patients and 115 IVIG-resistant KD patients. There were 137 KD patients and 152 febrile controls. The patients with acute KD have blood drawing before treated with globulin in most of studies (13/18). And the detection method was qRT-PCR in most of studies (12/18). Convalescent KD patients returned to normal and clinical symptoms disappeared. The specimen types were serum, plasma, platelets and PBMCs. We found that miR-92a-3p could be used as a biomarker in distinguishing KD-CAL patients from KD-NCAL patients and acute KD patients from convalescent KD patients, with the under the curve (AUC) of 0.816 (95% CI 0.669–0.962) and 0.748 (95% CI 0.634–0.861), respectively. The QUADAS-2 assessment indicated that all studies had a moderate to high risk of bias in patient selection and index tests, and all studies had a low risk of bias in reference standards and flow and timing. All included studies were deemed as low risk for applicability in the reference standard, and all included studies were deemed as “Unclear” in patient selection and index test. Most of the studies were not completely designed to be diagnostic accuracy studies, and some questions were not applicable. Therefore, the reviewers indicated this choice as “Unclear.” The results are shown in Figures 2A,B.

**Table 1:** The primary characteristics of the 12 included studies in this review.

Author	Country	control	Reference	Reference RNA	Specimen	Assay Method	Timing	case (male/female)	Age of Case (m)	Control (male/female)	Age of Control (m)	ncRNA	Expression
Guan YQ 2020 (27)	China	NCAL	AHA	Cel-miR-39-3P	Serum	qRT-PCR	Before treatment	24 (18/6)	3.80–11.00	25 (13/12)	6.00–54	MiR-145	Up-regulated
Chu MP 2015 (28)	China	NCAL	JCS	MiR-16	PBMC	RT-PCR	Before treatment	15	N/R	15	Unclear	MiR-130a	Up-regulated
Chu MP 2015 (28)	China	NCAL	JCS	MiR-16	PBMC	RT-PCR	Before treatment	15	N/R	15	Unclear	MiR-125b	Up-regulated
Jing FC 2021 (19)	China	NCAL	JCS	Hsa-miR-103a-3p	Serum	qRT-PCR	Before treatment	10 (8/2)	26.796 ± 21.864	10 (7/3)	41.496 ± 26.964	MiR-221-3p	N/R
Rong X 2017 (20)	China	NCAL	AHA	Cel-miR-39	Serum	RT-PCR	Before treatment	12 (11/1)	26.36 ± 18.06	33 (23/10)	24.6 ± 19.23	MiR-92a-3p	Up-regulated
Wang ZY 2019 (21)	China	NCAL	JCS	U6	PBMC	RT-PCR	Before treatment	25	N/R	25	N/R	MiR-937	Down-regulated
Chen RY 2020 (29)	China	RS	AHA	Cel-miR-39	Plasma	qRT-PCR	N/R	30	N/R	31	N/R	Linc01635	Up-regulated
Luo YP 2021 (12)	China	RS	JCS	MiR-16	Plasma	qRT-PCR	Before treatment	30 (16/14)	38.88 ± 27.96	30	N/R	MiR-133a	Up-regulated
Rong X 2017 (20)	China	RS	AHA	Cel-miR-39	Serum	RT-PCR	Before treatment	45 (34/11)	25.3 ± 12.6	30 (25/5)	25.3 ± 12.6	MiR-92a-3p	Up-regulated
Rong X 2018 (30)	China	RS	AHA	Cel-miR-39	Serum	qRT-PCR	Before treatment	34 (24/10)	32.79 ± 15.56	18 (11/7)	30.68 ± 27.18	MiR-27b	Up-regulated
Guan YQ 2020 (24)	China	FC	AHA	Cel-miR-39-3P	Serum	qRT-PCR	Before treatment	28 (20/8)	23.53 ± 15.63	28 (14/14)	43.89 ± 39.98	Mi-145, ALB	Up-regulated
Hu QQ 2020 (31)	China	FC	AHA	Cel-miR-39-3P	Serum	RT-PCR	Before treatment	30 (19/11)	26.00 ± 16.88	30 (16/14)	40.67 ± 34.09	MiR-223-3P CRP, ALT, Alb	–
Ning QQ 2020 (22)	China	FC	AHA	MiR-126-3P	Platelet	qRT-PCR	N/R	33	N/R	28	N/R	5 MiRNA + 6Clinical characteristics	–
Ning QQ 2021 (32)	China	FC	AHA	MiR-126-3P	Platelets	qRT-PCR	Before treatment	46	N/R	66	N/R	ESR, WBC, ALB, miR- 15a-5p and miR-941	–
Zhang W 2017 (23)	China	IVIG-responsive	AHA	U6	Serum	qRT-PCR	Within 24 h of KD diagnosis	34	N/R	68	N/R	MiR-20°C	Up-regulated
Zhang W 2017 (23)	China	IVIG-responsive	AHA	U6	Serum	qRT-PCR	within 24 h of KD diagnosis	34	N/R	68	N/R	MiR-371-5p	Up-regulated
Zhang W 2017 (23)	China	IVIG-responsive	AHA	U6	Serum	qRT-PCR	Within 24 h of KD diagnosis	34	N/R	68	N/R	MiR-20°c and MiR-371-5p	Up-regulated
Jing FC 2021 (19)	China	IVIG-responsive	JCS	Hsa-miR-103a-3p	Serum	qRT-PCR	Before treatment	13 (10/3)	28.188 ± 14.532	42 (27/15)	28.548 ± 20.976	MiR-221-3p	Up-regulated

AHA, American heart association; FC, febrile control; JCS, Japanese cardiology society; RS, recover stage; miR, micro RNA; Linc, long nocoding RNA; IVIG, intravenous immunoglobulin; N/R, not report.

**Table 2:** Main characteristics of the included studies.

Author	AUC	95%CI	Sensitivity	Specificity	TP	FP	FN	TN
Guan YQ 2020	0.731	0.586–0.879	75.00%	76.00%	18	6	6	19
Chu MP 2015	0.854	N/R	87.50%	83.30%	13	3	2	12
Chu MP 2015	0.750	N/R	77.50%	76.60%	12	4	3	11
Jing FC 2021	0.830	0.648–1.000	60.15%	100.00%	6	0	4	10
Rong X 2017	0.816	0.669–0.962	81.80%	66.70%	10	11	2	22
Wang ZY 2019	0.8986	0.761–0.990	77.50%	76.60%	19	6	6	19
Chen RY 2020	0.837	0.729–0.944	73.22%	96.90%	22	1	8	30
Luo YP 2021	0.860	0.760–0.950	86.67%	73.33%	26	8	4	22
Rong X 2017	0.748	0.634–0.861	62.10%	80.00%	28	6	17	24
Rong X 2018	0.824	0.702–0.946	91.40%	66.70%	31	6	3	12
Guan YQ 2020	0.847	0.747–0.947	92.90%	67.90%	26	9	2	19
Hu QQ 2020	0.853	0.756–0.951	80.00%	76.7%	24	7	6	23
Ning QQ 2020	0.930	N/R	86.20%	95.20%	28	1	5	27
Ning QQ 2021	0.975	N/R	93.50%	92.40%	43	5	3	61
Zhang W 2017	0.940	N/R	40.00%	91.60%	14	6	20	62
Zhang W 2017	0.940	N/R	40.00%	91.60%	14	6	20	62
Zhang W 2017	0.970	0.923–1.035	91.60%	83.30%	31	11	3	57
Jing FC 2021	0.811	0.672–0.951	76.86%	83.06%	10	7	3	35

AUC, area under the curve; TP, true positive; FP, false positive; FN, false negative; TN, true negative; N/R, not report.

### 3.2. Data synthesis and analysis

#### 3.2.1. The diagnostic performance of ncRNAs for KD-CAL

**Figure 3:**
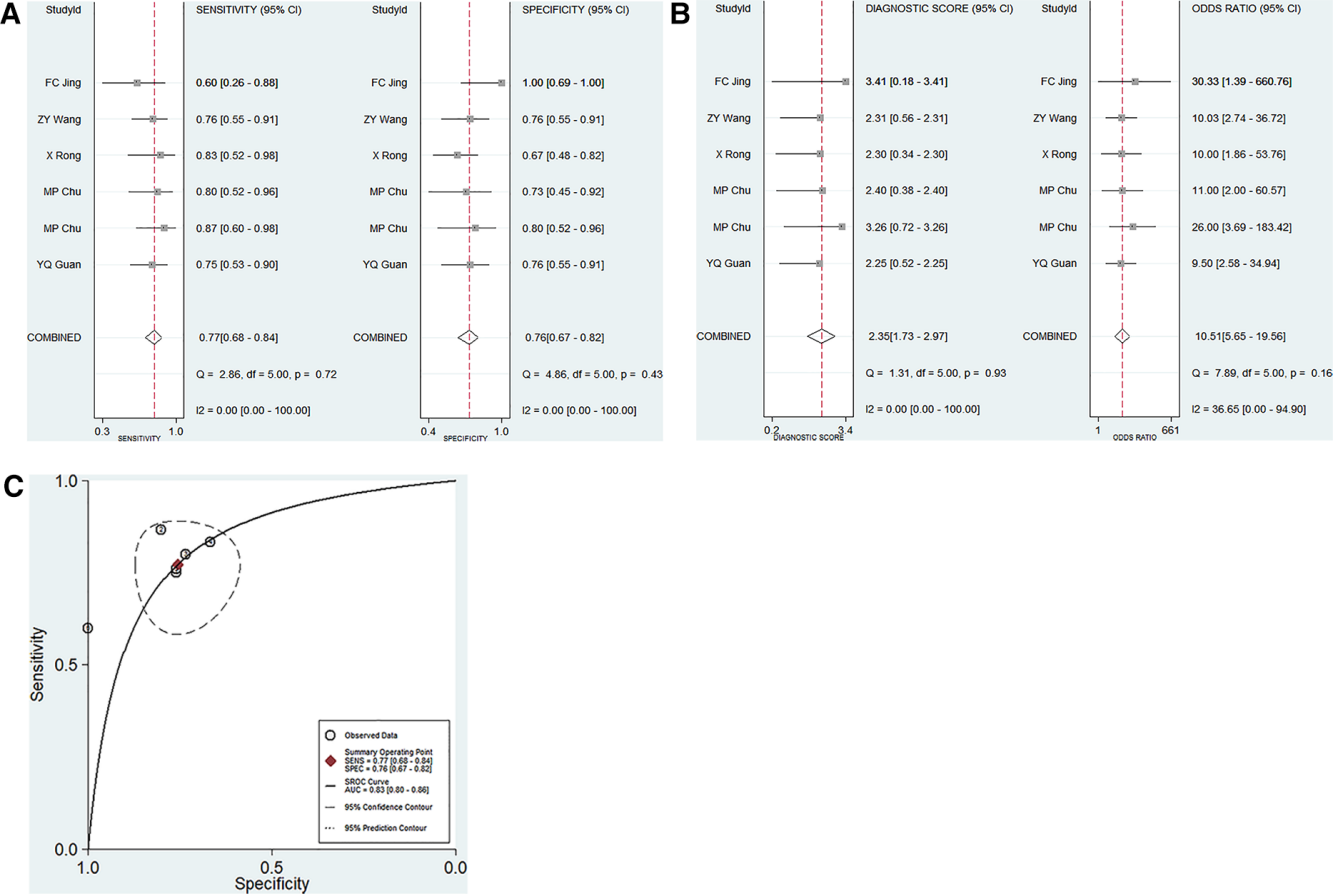
Pooled results of the studies on overall ncRNAs used in the diagnosis for KD-CAL among 6 studies included in the meta-analysis. (**A**) Pooled sensitivity and specificity; (**B**) Pooled DOR; (**C**) SROC.

A total of 5 articles including 6 studies on the diagnostic performance of ncRNAs in differentiating KD-CAL from KD-NCAL patients were included (Table 1). The results indicated that heterogeneity did not exist (*p* > 0.05, *I*^2^ = 0.0%), so a fixed effect model were used to synthesize the data. The pooled results were as follows: the calculated AUC value was 0.83 (0.80–0.86), pooled sensitivity was 0.79 (0.70–0.86), pooled specificity was 0.73 (0.65–0.81), positive likelihood ratio (PLR) was 3.17 (2.28–4.40), negative likelihood ratio (NLR) was 0.30 (0.21–0.44) and diagnostic odds ratio (DOR) was 10.48 (5.44–20.20), which indicated that ncRNAs have good value in differentiating KD-CAL from KD-NCAL patients (Figures 3A–C).

#### 3.2.2. The diagnostic performance of ncRNAs combined with other laboratory indexes for KD

**Figure 4:**
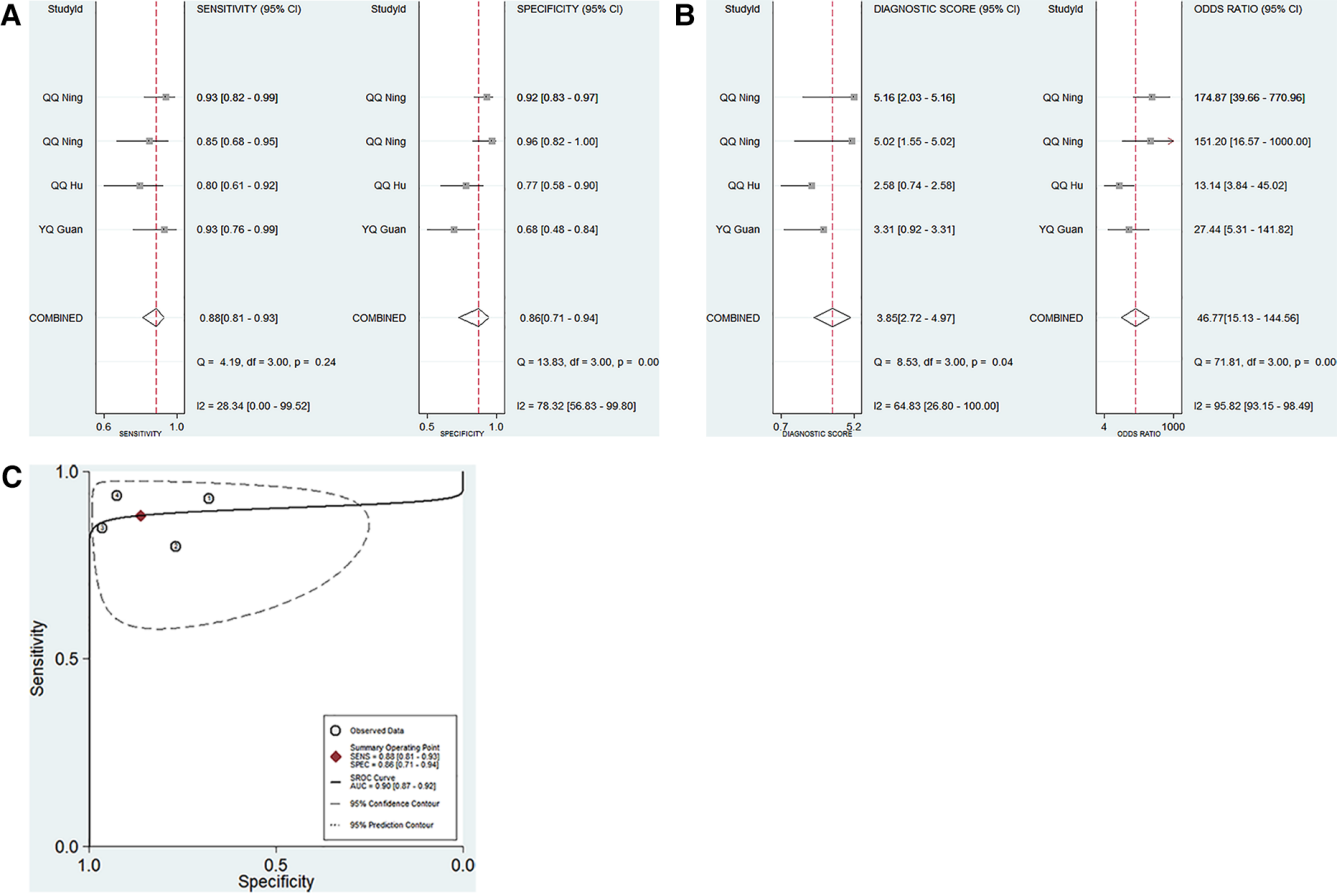
Pooled results of the studies on ncRNAs combined with other laboratory indexes for KD among 4 studies included in the meta-analysis. (**A**) Pooled sensitivity and specificity; (**B**) Pooled DOR; (**C**) SROC.

Four studies focused on the diagnostic performance of ncRNAs combined with other laboratory indexes in KD (Table 1). This study showed that no significant heterogeneity existed among the included studies (*p* > 0.05, *I*^2^ = 28.34%). Therefore, a fixed effect model was used to calculate the results. The pooled results were as follows: the calculated AUC value was 0.90 (0.87–0.92), pooled sensitivity was 0.88 (0.81–0.93), pooled specificity was 0.86 (0.71–0.94), DOR was 46.77 (15.13–144.56), PLR was 6.38 (2.84–14.34), and the NLR was 0.14 (0.08–0.23). These results showed that ncRNAs combined with other laboratory indexes have a great diagnostic performance. Compared with our research group's previous study results, the diagnostic performance of ncRNAs combined with other laboratory indices was better than that of only ncRNAs in KD patients (Figures 4A–C).

#### 3.2.3. The diagnostic performance of ncRNAs in differentiating acute KD patients from convalescent KD patients

**Figure 5:**
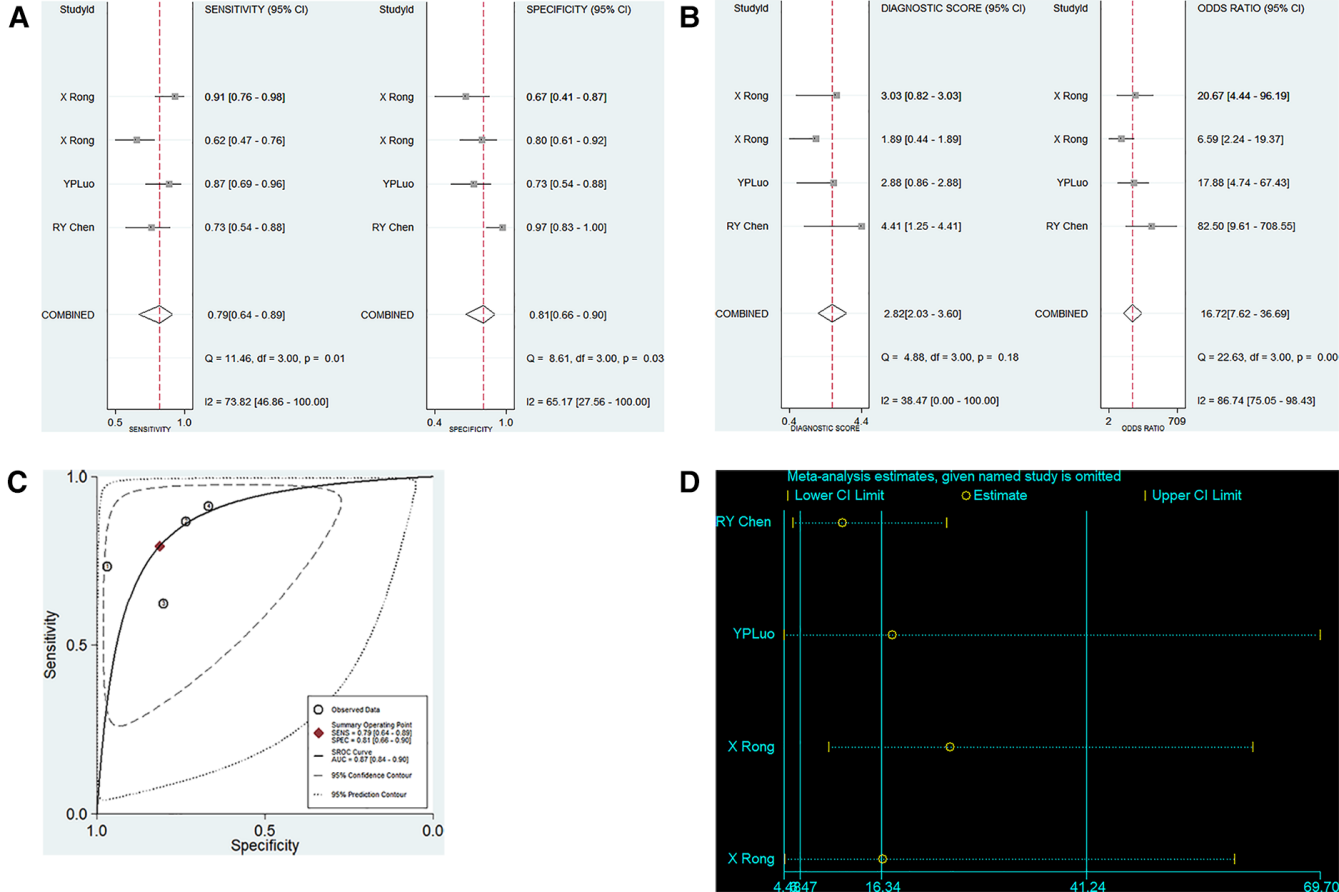
Pooled results of the studies on ncRNAs differentiating acute KD patients from convalescent KD patients among 4 studies included in the meta-analysis. (**A**) Pooled sensitivity and specificity; (**B**) Pooled DOR; (**C**) SROC. (**D**). Sensitivity analysis of the results of the meta-analysis for ncRNAs differentiating acute KD patients from convalescent KD patients.

A total of 4 studies on the diagnostic performance of ncRNAs in differentiating acute KD patients from convalescent KD patients were conducted (Table 1). The pooled estimation showed significant heterogeneity in the two groups (*p* < 0.05, *I*^2^ = 73.82%), so the random effect model was used to calculate the results. The pooled results were as follows: the calculated AUC value was 0.87 (0.84–0.90), pooled sensitivity was 0.79 (0.64–0.89), pooled specificity was 0.81 (0.66–0.90), PLR was 4.23 (2.38–7.49), NLR was 0.25 (0.15–0.44) and DOR was 16.72 (7.62–36.69). The results indicated that ncRNAs have good diagnostic value in differentiating acute KD patients from convalescent KD patients (Figures 5A–C).

We conducted a sensitivity analysis to assess the contribution of each study to the pooled estimate. However, no exclusions altered the magnitude of the pooled estimate, which further confirmed the validity of the study (Figure 5D).

#### 3.2.4. The diagnostic performance of ncRNAs in differentiating IVIG-resistant KD patients from IVIG-responsive KD patients

**Figure 6:**
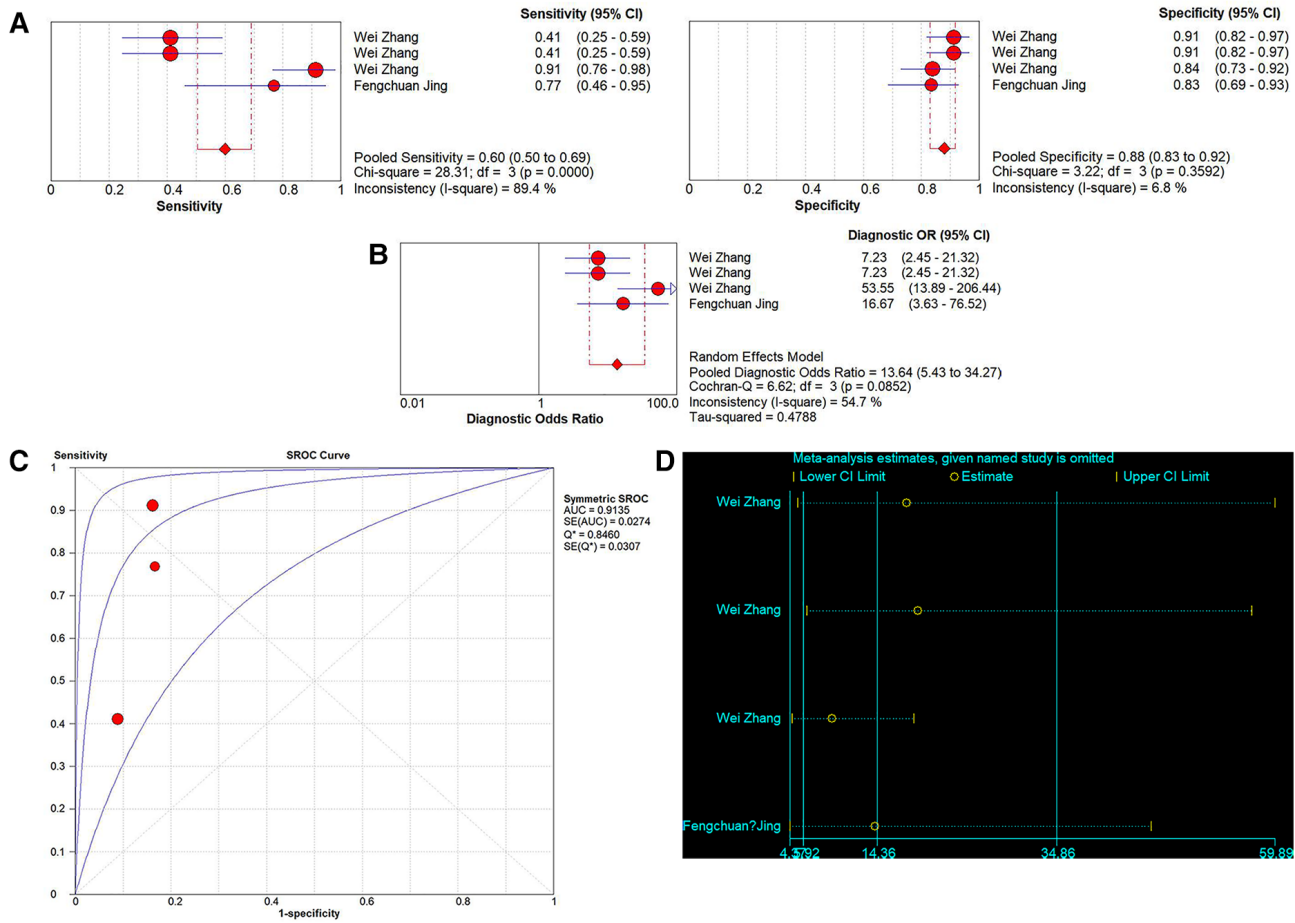
Pooled results of the studies on ncRNAs differentiating IVIG-resistant KD patients from IVIG-responsive KD patients among 4 studies. (**A**) Pooled sensitivity and specificity; (**B**) Pooled DOR; (**C**) SROC. (**D**) Sensitivity analysis of the results of the meta-analysis for IVIG-resistant KD patients from IVIG-responsive KD patients.

A total of 4 studies on the diagnostic performance of ncRNAs in differentiating IVIG-resistant KD patients from IVIG-responsive KD patients were conducted (Table 1). The results showed significant heterogeneity in the two groups (*p* < 0.05, *I*^2^ = 89.4%), so the random effect model was used to calculate the results. The pooled results were as follows: the calculated AUC value was 0.9135 ± 0.0307, pooled sensitivity was 0.60 (0.50–0.69), pooled specificity was 0.88 (0.83–0.92), PLR was 5.04 (3.52–7.21), NLR was 0.41 (0.22–0.76) and DOR was 13.64 (5.43–34.27). The results indicated that ncRNAs have great diagnostic values in differentiating IVIG-resistant KD patients from IVIG-responsive KD patients (Figures 6A–C).

Sensitivity analysis was used to assess the contribution of each study to the pooled estimate. However, the results showed that no exclusions altered the magnitude of the pooled estimate, which further confirmed the validity of the study (Figure 6D).

### 3.3. Publication bias

**Figure 7:**
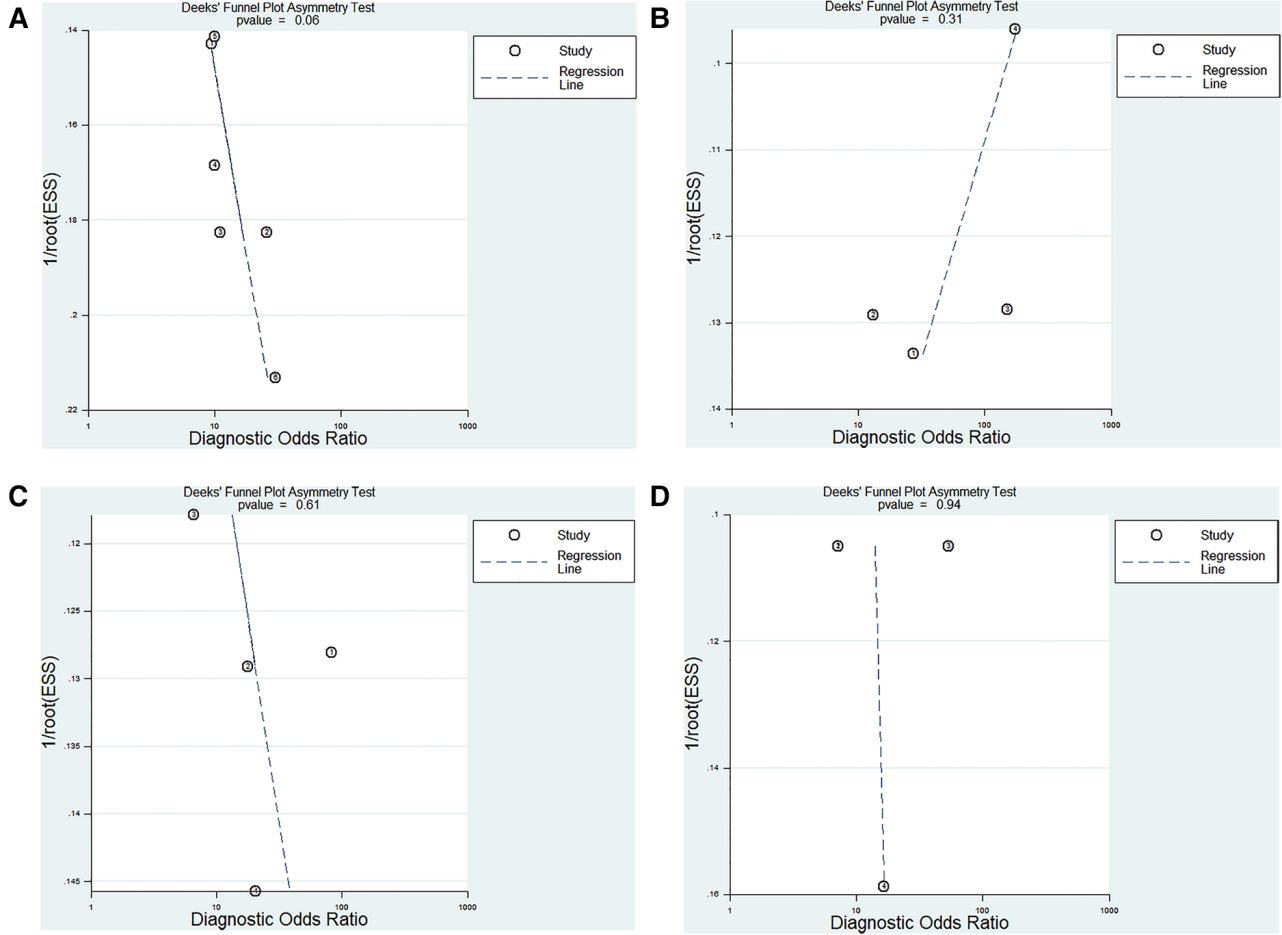
Deeks funnel plot evaluating the potential publication bias of the included studies. (**A**) ncRNAs diagnosis for KD-CAL; (**B**) ncRNAs combined with other laboratory indexes for KD; (**C**) ncRNAs differentiating acute KD patients from convalescent KD patients; (**D**) ncRNAs differentiating IVIG-resistant KD patients from IVIG-responsive KD patients.

The Deeks test was performed to assess publication bias. The results showed that publication bias did not exist in our study (Figures 7A–D).

## 4. Discussion

A previous meta-analysis explored the diagnostic value of miRNAs in KD patients, which indicated that the summary AUC value was 0.8302 ± 0.0402, the pooled sensitivity was 0.70 (95% CI, 0.66–0.74), and the pooled specificity was 0.87 (95% CI, 0.83–0.90) (18). That study conducted a conclusion that further research should attempt to find the diagnostic values of miRNAs combined with other laboratory tests in KD patients, which may provide a better detection method for KD. In this study, we conducted a meta-analysis to identify the diagnostic value of miRNAs combined with other clinical laboratory tests in KD. To the best of our knowledge, this is the first systematic review and meta-analysis to comprehensively analyze the diagnostic accuracy of ncRNAs, mainly miRNAs, in differentiating KD-CAL from KD-NCAL, acute KD patients from convalescent KD patients, IVIG-resistant KD patients from IVIG-responsive KD patients and the diagnostic accuracy of miRNAs combined with other clinical laboratory tests in KD.

The CAL are the primary cause of acquired heart diseases in children (33). Timing therapy could reduce the risk of CAL from 25% to approximately 4% and reduce cost and hospitalization time (1). Therefore, early diagnosis of KD-CAL is very important. A total of 6 relevant studies assessing 101 KD-CAL patients were included in this meta-analysis. Our results suggested that miRNAs have a good diagnostic accuracy for detecting KD-CAL and could be used as biomarkers for detecting KD-CAL patients. Our results showed that ncRNAs were upregulated in acute KD patients and decreased in convalescent KD patients. The pooled results were as follows: the calculated AUC was 0.87 (0.84–0.90), the pooled sensitivity was 0.79 (0.64–0.89), and the pooled specificity was 0.81 (0.66–0.90). This showed that ncRNAs have a good diagnostic performance in differentiating acute KD patients from convalescent KD patients. The results suggested that the diagnostic accuracy of miRNAs combined with other clinical laboratory tests was higher than that of miRNAs alone, with a calculated AUC value of 0.90 (0.87–0.92), a pooled sensitivity of 0.88 (0.81–0.93) and a pooled specificity of 0.86 (0.71–0.94) VS a summary AUC value of 0.8302 ± 0.0402, a pooled sensitivity of 0.70 (0.66–0.74) and a pooled specificity of 0.87 (0.83–0.90) (18).

Wu Gang (17) conducted a systematic review of the neutrophil-to-lymphocyte ratio as a biomarker for predicting IVIG-resistant KD. None of the systematic reviews investigated if ncRNAs could be used as biomarkers in IVIG-resistant KD. Jing Fengchuan and Zhang Wei (19, 23) suggested that miR-221-3p, miR-200c and miR-371-5p could be biomarkers for the diagnosis of IVIG-resistant KD, with AUC of 0.811, 0.94 and 0.89, respectively. Our results showed that ncRNAs have great diagnostic values in differentiating IVIG-resistant KD from IVIG-responsive KD patients. However, only 2 articles were searched, including 4 original studies, which restricts the ability to obtain a valid conclusion. More original research is needed to identify the diagnostic accuracy for IVIG-resistant KD. Zheng XL (34) conducted a systematic review and meta-analysis indicating that the incidence of CAL in the IVIG-resistant group was higher than that in the IVIG-responsive group. Many studies have suggested that IVIG resistance is deeply associated with the occurrence of CAL (35–37). However, the molecular mechanisms of IVIG and CAL in KD are still unclear (35). Therefore, more well-designed studies are needed to explore the incidence of CAL in IVIG-resistant patients.

The results from this meta-analysis and our previous results suggested that ncRNAs, mainly miRNAs, could be used as new biomarkers for differentiating acute KD patients from healthy patients, febrile patients and convalescent KD analysis. We conducted a sensitivity analysis and publication bias, and we did not find any sources of heterogeneity. MiRNAs are readily detectable in the plasma, and the results of our study suggested that miRNAs are suitable as ideal biomarkers (13).

Research on ncRNA-based biomarkers is still in the early stage of development and still not ready for clinical application as a diagnostic test for KD. The epidemiology of KD varies with ethnicity (38). Most studies were conducted in small populations, and the results were not reproducible across populations and ethnicities. These studies need to be validated in other geographic populations and larger cohorts. A large number of studies are supposed to identify a ncRNA, miRNA or lncRNA, determining whether the specimen is serum, plasma, PBMC, platelet or other non-invasive biomarker, such as urine, could be used as a diagnostic biomarker in differentiating KD-CAL/KD-NCAL, acute/convalescent phase of KD, IVIG resistant/responsive KD patients and ncRNAs combined with other laboratory indicators to diagnose KD. Further studies should be conducted to explore the timing of blood collection. It was recommended that blood was taken before IVIG was treated.

Undoubtedly, this meta-analysis has some limitations. First, the limited number of included studies may have resulted in unstable results. It is necessary to include more original articles to validate the results. Second, only Chinese publications were included, which restricts the ability to generalize the findings to the rest of the world. Third, inconsistent diagnosis criteria of KD-CAL between different studies may lead to bias. Fourth, the included studies had a moderate to high risk of bias in patient selection and index tests, necessitating further research to improve this aspect.

In conclusion, this meta-analysis indicated that ncRNAs have good diagnostic values in differentiating KD-CAL from KD-NCAL, acute KD patients from convalescent KD patients, IVIG-resistant KD from IVIG-responsive KD patients and have a higher diagnostic accuracy when miRNAs combined with other clinical laboratory tests than miRNAs alone used to detect for KD. However, the number of original studies was too small to make a definitive conclusion. Research on ncRNAs is still in its infancy and is not yet a diagnostic test for KD. These results cannot be replicated across different populations and ethnicities, which need to be validated in different populations when determining their usefulness in the diagnosis of KD. The biomarker is a pointer to the diagnosis of KD but it is still in it's early stage, and much more work needs to be done before it becomes a powerful laboratory diagnostic test. More well-designed clinical and experimental studies are needed to explore the diagnostic value of ncRNAs for KD and the cause and mechanisms.

## Data Availability

The original contributions presented in the study are included in the article/Supplementary Material, further inquiries can be directed to the corresponding author/s.
